# Delirium Tremens in an Infant

**Published:** 1846-12

**Authors:** 


*Delirium Tremens in an Infant.*—A little boy, five years of age, swallowed by mistake a large quantity of brandy. Vomiting speedily followed, and he passed a restless night, sleeping only toward morning. On awaking, it was observed that he had tremor of the hands, and that he could not hold a cup steadily. Convulsions with cramps ensued. The pulse was slow, the look timid, the pupils dilated, and the countenance pale. Delirium supervened, and there was dysuria with great thirst. A cataplasm was applied to the abdomen, and calomel and jalap were administered. The symptoms abated about the middle of the day; but towards evening there was a return of the tremor, with other nervous symptoms. An opiate was exhibited, from the effects of which the child slept soundly, and on awaking the whole of the symptoms had disappeared.—*Gaz. des Hôp.—Ibid.*

